# Puberty timing associated with diabetes, cardiovascular disease and also diverse health outcomes in men and women: the UK Biobank study

**DOI:** 10.1038/srep11208

**Published:** 2015-06-18

**Authors:** Felix R Day, Cathy E Elks, Anna Murray, Ken K Ong, John R.B. Perry

**Affiliations:** 1 MRC Epidemiology Unit, University of Cambridge School of Clinical Medicine, Institute of Metabolic Science, Cambridge Biomedical Campus Box 285, Cambridge, CB2 0QQ; 2 Genetics of Complex Traits, University of Exeter Medical School, RILD Level 3, Royal Devon & Exeter Hospital, Barrack Road, Exeter, EX2 5DW; 3 Department of Paediatrics, University of Cambridge, Cambridge Biomedical Campus, Cambridge, CB2 0QQ

## Abstract

Early puberty timing is associated with higher risks for type 2 diabetes (T2D) and cardiovascular disease in women and therefore represents a potential target for early preventive interventions. We characterised the range of diseases and other adverse health outcomes associated with early or late puberty timing in men and women in the very large UK Biobank study. Recalled puberty timing and past/current diseases were self-reported by questionnaire. We limited analyses to individuals of White ethnicity (250,037 women; 197,714 men) and to disease outcomes with at least 500 cases (~0·2% prevalence) and we applied stringent correction for multiple testing (corrected threshold P < 7.48 × 10^–5^). In models adjusted for socioeconomic position and adiposity/body composition variables, both in women and men separately, earlier puberty timing was associated with higher risks for angina, hypertension and T2D. Furthermore, compared to the median/average group, earlier or later puberty timing in women or men was associated with higher risks for 48 adverse outcomes, across a range of cancers, cardio-metabolic, gynaecological/obstetric, gastrointestinal, musculoskeletal, and neuro-cognitive categories. Notably, both early and late menarche were associated with higher risks for early natural menopause in women. Puberty timing in both men and women appears to have a profound impact on later health.

Puberty represents the biological and psychological transition from childhood to adulthood. Its timing shows remarkably wide between-individual variation, with the normal age at onset of puberty ranging from 8 to 13 years in girls, and from 9 to 14 years in boys^1^,^2^. Menarche, the onset of first menstruation in girls represents a distinct event in puberty. It is reasonably well-recalled into adulthood and has therefore been included in many epidemiological studies^3^, with growing evidence that early age at menarche is associated with higher risks for subsequent obesity, type 2 diabetes (T2D)^4^ and cardiovascular disease^5^. Other reported associations with early menarche include higher risks for breast cancer^6^ and all-cause mortality^7^ and there are less consistent links described with depression and low bone mineral density^8^,^9^. In men, the evidence for such associations is sparse due to the lack of a convenient measure of puberty timing. Some adverse health associations with early puberty timing in men have been described, based on timing of voice breaking, with later adiposity^10^ and blood pressure^11^. Similar to menarche in girls, voice breaking in boys represents a distinct event of late puberty^12^ and, at least when prospectively assessed, shows reasonable correlation with other physical changes of puberty and has been shown to be a sensitive measure of secular trends in puberty timing and its relationships with childhood growth^10^,^13^.

The UK Biobank study provides the opportunity to study the correlates of disease on an unprecedented scale^14^. Comprising approximately 500,000 UK individuals aged predominantly between 40-70 years, extensive baseline data have been recorded on a wide range of prevalent diseases. In this study, we performed the largest scale assessment to date of the potential impact of puberty timing on risks of adverse health outcomes. We robustly confirm the associations between early puberty timing and T2D/cardiovascular disease in women, we describe very similar associations with early puberty timing in men, and we implicate new links between puberty timing in both men and women and a wide range of health outcomes.

## Methods

### Population and study design

The UK Biobank study design has been reported^14^. Briefly, all people aged 40–69 years who were registered with the National Health Service and living up to ~25 miles from one of the 22 study assessment centres were invited to participate in 2006-10. Overall, about 9.2 million invitations were mailed in order to recruit 503,325 participants (i.e. a response rate of 5.47%)^15^. Extensive self-reported baseline data were collected by questionnaire, in addition to anthropometric assessments. Age at menarche in women was self-reported in whole years and age at voice breaking in men reported as a categorical variable, where participants were asked whether their voice broke at an age “younger”, “about average” or “older” than their peers. For the current analysis, individuals of non-white ethnicity (N = 29,819) were excluded to avoid potential confounding effects of ancestry on puberty timing and disease risks^3^. Furthermore, we excluded women who did not report age at menarche (N = 7,318), or reported age at menarche at very extreme ages: <8 years (N = 29) or >19 years (N = 121), and we excluded men who did not report timing of voice breaking (N = 17,617). All participants provided informed written consent, the study was approved by the National Research Ethics Service Committee North West – Haydock, and all study procedures were performed in accordance with the World Medical Association Declaration of Helsinki ethical principles for medical research.

### Adverse health outcomes

Past or current diseases were self-reported in response to the question “Has a doctor ever told you that you have had any of the following conditions? (You can select more than one answer)”. To ensure good discrimination between medical conditions, the data were collected using a computer-assisted personal interview (CAPI), administered by trained interviewers. Twelve other adverse health outcomes were generated by re-classification of questionnaire data or objective measurements made at the baseline visit. Seven adverse outcomes were generated in both men and women; these were: *short stature* (defined as the lowest 5% of measured height, separately for men and women); *obesity* (BMI > 30 kg/m^2^ based on measured height and weight); *low intelligence* (scores of 2 or less out of a possible 13 on the UK Biobank fluid intelligence test, ~3·8% of the study sample); *low FEV1* (low forced expiratory volume in 1 second, the lowest 5% of residuals for FEV1 from a model with height and sex as covariates), *low trauma fracture* (history of any fracture resulting from a simple fall); *poor sleep* (less than five hours sleep; compared to those reporting 8 hours); and *overall poor health* (those who answered “Poor” to the question “In general how would you rate your overall health?”). The five adverse outcomes specific to women were: *stillbirth*, *low birth weight of first child* (<5·5 pounds or <2·49 kg); *oophorectomy*, *hysterectomy*, and *early natural menopause* (defined as menopause occurring before age 45, without a prior hysterectomy or oophorectomy, and not taking hormone replacement therapy at the time of menopause)^16^.

Comparator groups were identified separately for each outcome. In general, any participant who did not report a specific disease was considered to be a non-case for that disease. For T2D, we excluded from the analysis model any case who might have type 1 diabetes (based on age at diagnosis ≤ 35 years, insulin use within 1 year of diagnosis, or were diagnosed less than one year prior to their UK Biobank assessment). Where adverse outcomes were derived from re-classification of other data, in general the comparator group comprised all men or women who provided higher (i.e. less adverse) responses/measurements, except where stated above.

To provide sufficient power to meet our conservative significance threshold, we considered only those diseases/outcomes with least 500 cases in either sex (~0·2% prevalence). In total, we considered 128 diseases plus 12 other adverse outcomes in women, and 112 diseases plus 7 other adverse outcomes in men.

### Statistical analysis

Separate logistic regression models in each sex were performed to test the associations between puberty timing and each outcome. Age at menarche in women was analysed in linear models and also in two categorical models, which compared the earliest approximate quintile (8–11 years inclusive, N = 50,405) or oldest approximate quintile (15–19 years inclusive, N = 41,338) to the median (13 years, N = 61,216). Age at voice breaking in men was analysed in only two categorical models, comparing either the “relatively younger” or the “relatively older” voice breaking group to the “about average” group.

Baseline models included birth year, age, and age-squared, to account for potential confounding effects of the secular changes in puberty timing. Further adjusted models were also performed to account for the potential confounding and mediating effects of socio-economic position (SEP) and adiposity/body composition. To enable comprehensive adjustments without invalidating our models due to co-linearity, we performed a two-stage analysis. First, we calculated the principle components for all available *‘adiposity/body composition’-*related variables (BMI, hip circumference and waist circumference, – measured by trained assessment centre staff; and weight, body fat percent, trunk fat free mass, trunk fat mass, whole body fat free mass, whole body fat mass and whole body water mass – all estimated by the Tanita BC418MA electrical bioimpedance analyser) or the available *‘SEP’-*related variables (alcohol intake; education – 8 dummy variables for different levels of qualification, maternal smoking, reported income level, smoking – ever and current, Townsend index of deprivation). We then included in our adjusted logistic models the top principle components (explaining in each case over 99% of the variance) for adiposity/body composition (5 principle components) and SEP (11 principle components). Where ‘Obesity’ was the outcome, the adjusted models included only the principal components for SEP.

A conservative multiple test-corrected threshold of P < 7·48 × 10^−5^ was used to identify significant disease associations; this value represented P = 0·05 divided by the total number of tests performed (140 outcomes × 3 models in women, plus 119 outcomes × 2 models in men).

## Results

Age at menarche within the range 8–19 years (mean 13.0, SD 1.6) was self-reported in completed whole years by 250,037 women of White ethnicity: 50,405 women had early menarche (8–11 years inclusive, 20·2%), 41,338 women had late menarche (15–19 years inclusive, 16·5%), and 61,216 women reported having menarche at the median age (13 years, 24·5%). Age at voice breaking was self-reported in 197,714 men of White ethnicity: 8,550 men (4·3%) reported they were “relatively younger”, 11,723 men (5·9%) reported they were “relatively older”, and the remainder reported they were “about average”.

### Menarche timing and adverse health outcomes in women

**Figure 1:**
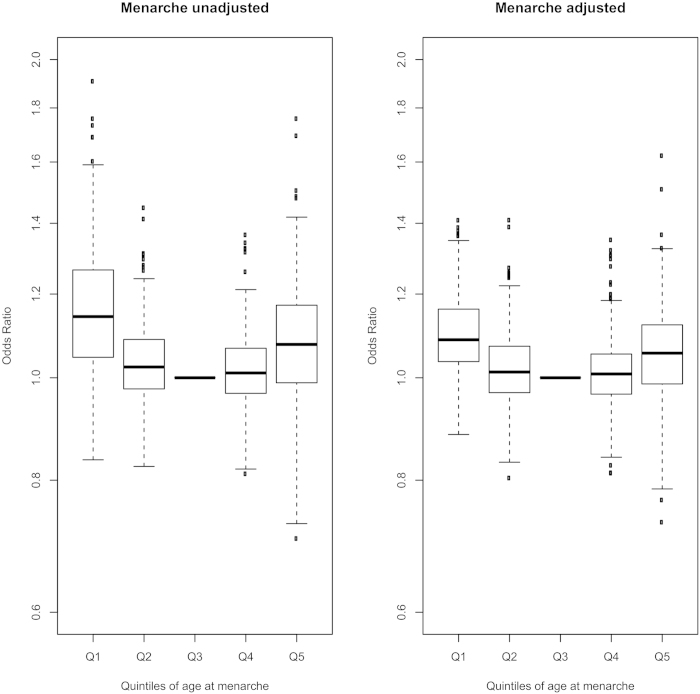
Distribution of effect sizes by quintile of menarche age for all of the 140 tested diseases and adverse health outcomes in women. The odds ratio for each disease for each quintile is calculated relative to the middle quintile (Q3). The box in each plot represents the inter quartile range (IQR) of the effect estimates the whiskers represent the range of effects that are 1.5 × IQR beyond the IQR, odds ratios for disease more extreme than this are plotted as individual points.

In linear models, age at menarche in women was associated with 26 adverse health outcomes at P-values below our multiple test-corrected significance threshold (P < 7·48 × 10^−5^). These included several disease associations reported in previous studies, such as T2D (OR: 0·87 per year later menarche, 95% CI: 0·85–0·88, P < 1 × 10^−15^), obesity (OR: 0·83, 0·83–0·84, P < 1 × 10^−15^), and breast cancer (OR 0·97, 0·95–0·98, P = 4.3 × 10^−8^). They also included several novel adverse health outcome associations across a wide range of gynaecological/obstetric, gastrointestinal, musculoskeletal, and neuro-cognitive categories (Table 1, Fig. 1, Supplementary table 1).

**Figure 2:**
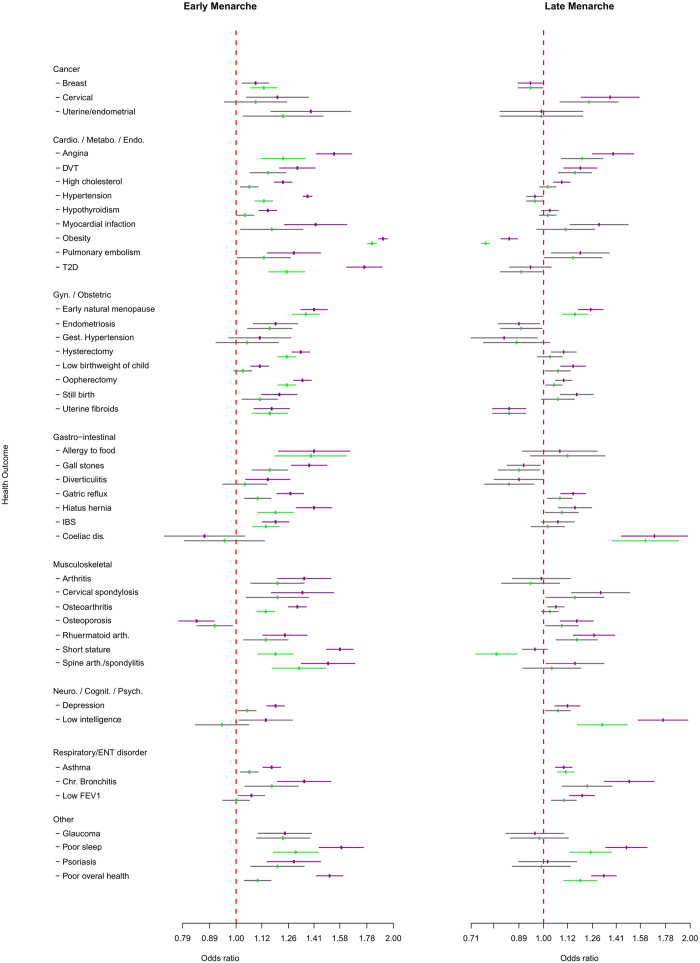
Associations between early or late menarche timing and adverse health outcomes in women. Displayed outcomes showed study-wise significant (P < 7.48 × 10^-5^) associations in any model (linear, early or late menarche). Effect estimates (± 95% CI) are shown for early (the earliest quintile) or late (the latest quintile) menarche groups compared to the middle quintile (reference group). Purple centres (with purple error bars) indicate significant associations from baseline models (adjusted for birth year, age and age squared). Green centres (with green error bars) indicate significant associations from models adjusted for socio-economic and adiposity/body composition variables (except for Obesity). Grey error bars indicate associations that did not reach study-wise significance.

Consideration of categorical early/late menarche models increased the number of associated adverse health outcomes to 42 (33 for early menarche, 19 for late menarche, and 25 in linear trend models). Remarkably, in all of the categorical associations, both early and late menarche groups were associated with *higher* risks of adverse outcomes compared to the median quintile group, with the exception of osteoporosis (lower in early menarche), obesity (lower in late menarche), and uterine fibroids (lower in late menarche) (Figs 1 and 2).

Following adjustment for potential confounding and mediation by SEP and adiposity/body composition, associations remained study-wise significant with 14 adverse health outcomes for early menarche, 8 for late menarche, and 14 in linear trend models, indicating possible direct links with menarche timing independent of adiposity and SEP. These included notable novel associations for early menarche with higher risks for: early natural menopause, uterine fibroids, hiatus hernia, osteoarthritis, and poor sleep quality; and for late menarche with higher risks for: early natural menopause, malabsorption/coeliac disease, low intelligence, asthma, poor overall health, and poor sleep. Endometriosis also retained significance in the adjusted linear model. (Table 1, Fig. 1).

### Voice breaking and adverse health outcomes in men

**Figure 3:**
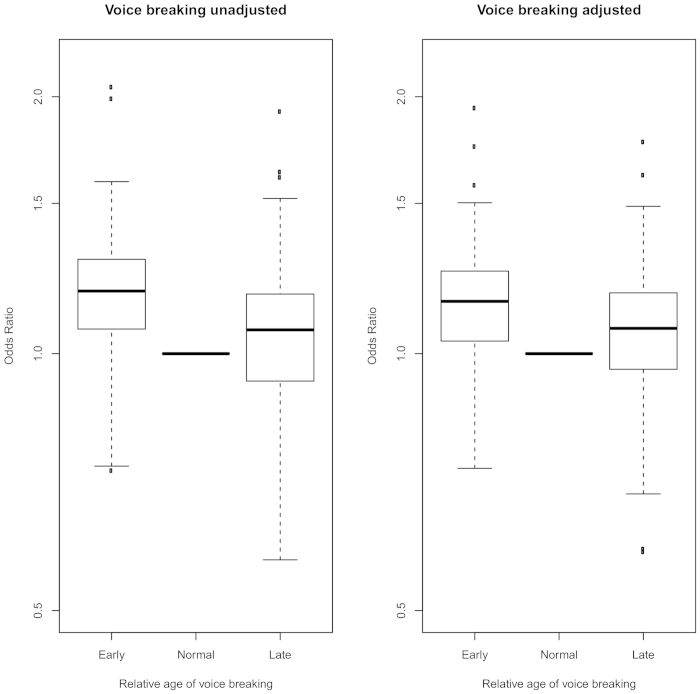
Distribution of effect sizes for early or late relative age at voice breaking for all 119 tested diseases and adverse health outcomes in men. The odds ratio for each disease for those with early or late voice breaking is calculated relative to the group with a normal age at voice breaking. The box in each plot represents the inter quartile range (IQR) of the effect estimates, the whiskers represent the range of effects that are 1.5 × IQR beyond the IQR, outliers more extreme than this are plotted as individual points.

**Figure 4:**
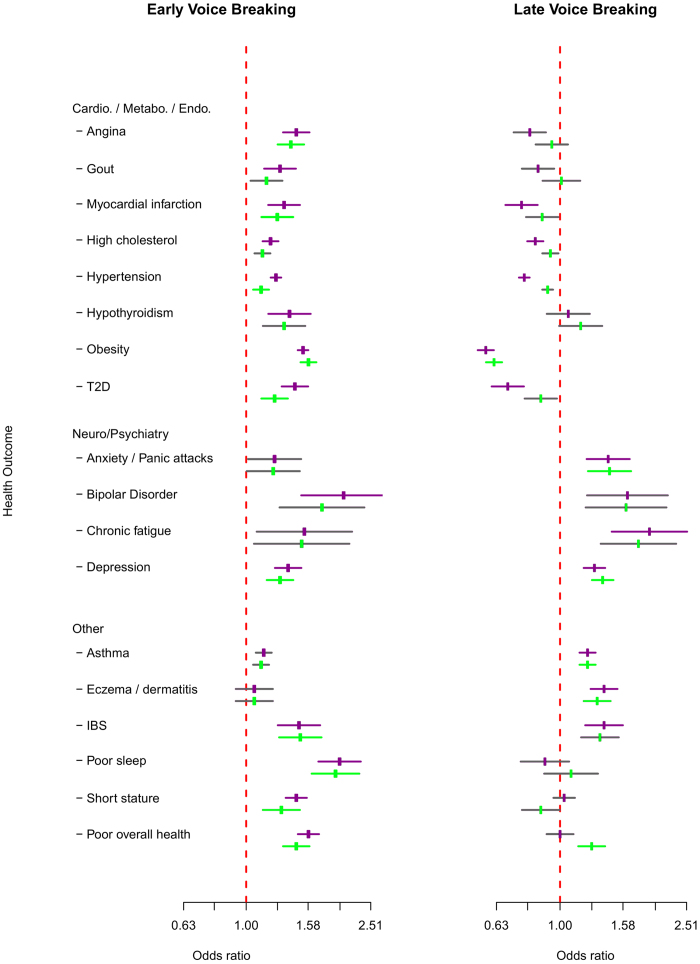
Associations between early or late voice breaking and adverse health outcomes in men. Displayed outcomes showed study-wise significant (P < 7.48 × 10^-5^) associations in any model (early or late). Effect estimates (± 95% CI) are shown for early or late voice breaking groups compared to the “about normal age” reference group). Purple centres (with purple error bars) indicate significant associations from baseline models (adjusted for birth year, age and age squared). Green centres (with green error bars) indicate significant associations from models adjusted for socio-economic and adiposity/body composition variables (except for Obesity). Grey error bars indicate associations that did not reach study-wise significance.

Relatively younger (versus about average) voice breaking in men was associated with 14 adverse outcomes and relatively older voice breaking was associated with 11 adverse outcomes (Table 2, Fig. 1). Younger voice breaking was invariably associated with *higher* risks of adverse outcomes, including several cardiovascular/metabolic and psychiatric outcomes. In contrast, older voice breaking appeared to be protective against cardiovascular/metabolic outcomes, but was associated with higher risks for other adverse outcomes. Apparent “U” shaped associations were seen for relative voice breaking with irritable bowel syndrome, asthma and some psychiatric outcomes (Table 2, Figs 3 and 4, Supplementary table 2).

Following adjustment for potential confounding and mediation by SEP and adiposity/body composition, associations remained significant with 9 adverse outcomes for younger voice breaking, indicating possible direct links between early puberty timing in men and higher risks for: cardiovascular/metabolic outcomes (angina, heart attack, hypertension, T2D and obesity), irritable bowel syndrome, depression, poor overall health and poor sleep. In contrast, adjustment for SEP and adiposity/body composition attenuated the apparent protective effects of older voice breaking on cardiovascular/metabolic outcomes, but there remained higher risks for: anxiety/panic attacks, depression, asthma, and eczema/dermatitis, and the association with higher risk of poor overall health was revealed.

### Overlap between disease associations in men and women

In adjusted models, both in women and men separately, earlier puberty timing was associated with higher risks for angina, hypertension and T2D, while later puberty was associated with higher risks for asthma and self-rated poor overall health.

When we considered associations with weaker evidence of association, all of the 14 diseases that were robustly associated with younger voice breaking in men (at P < 7·48 × 10^−5^ in Table 2) showed at least nominal associations (P < 0·05) with early menarche in women; all showed concordant directions of effect, except for bipolar disorder (Supplementary table 1). Findings were less concordant for older voice breaking in men: although 9/11 diseases showed at least nominal association with menarche timing in women, only five showed concordant directions of effect (hypertension, obesity, anxiety/panic attacks, depression and asthma). A reciprocal lookup in men of the outcomes that were robustly associated with early menarche revealed that 20 out of 27 possible outcomes showed at least nominal associations with younger voice breaking in men, all with concordant directions of effect. Findings were less concordant for the outcomes associated with later menarche in women: eight of the possible 15 outcomes showed at least nominal associations with older voice breaking in men; but only five showed concordant directions of effect (obesity, osteoporosis, depression, asthma, emphysema) (Supplementary table 1).

## Discussion

This very large study of half a million people has enabled us to comprehensively assess the potential impact of puberty timing on the risks of later life disease and other adverse health outcomes. We adopted a conservative statistical approach to provide robust confirmation that early menarche in women is associated with higher risks for T2D and cardiovascular disease. We also confirmed the linear associations between increasing age at menarche and higher risks for osteoporosis^17^ but lower risks for breast cancer^6^, which are both likely consequences of altered duration of exposure to reproductive levels of sex hormones. Furthermore, our findings substantially expand the range of associated adverse health outcomes, highlighting many with little or no prior link to age at menarche. These diseases span across a broad set of biological pathways, including gynaecological, gastrointestinal, musculoskeletal, neurological, psychiatric and respiratory conditions. The impact of puberty timing on disease risk is also extended to men, in whom we demonstrate for the first time associations with T2D and cardiovascular disease risks, concordant with those reported in women^5^.

The main limitation of our study is the cross-sectional design with reliance on self-reported medical histories and recalled measures of the exposures. Age at menarche can be recalled by women with moderate accuracy, with correlations between prospective and recalled measures several decades later ranging r = 0·66 to 0·80^18^,^19^ and the range and distributions of age at menarche were similar here as in previous large-scale studies^4^,^20^. Timing of voice breaking in men, when prospectively assessed, has been shown to be an accurate non-invasive measure of pubertal maturation^10^, but to our knowledge has not been validated when reported by recall in adult life. Our concordant findings and similar apparent effect sizes to those previously reported from prospective studies for blood pressure in men^11^ and for T2D^4^, cardiovascular disease^21^ and breast cancer^6^ in women indicates that the error in recalling these puberty timing exposures does not appear to be differentially allocated by disease status.

Other limitations exist as a consequence of our cross-sectional study design, including healthy selection bias, and the possibility of reverse-causality. Given that disease processes can begin long before clinical presentation or diagnosis, it remains possible that even childhood events could be influenced by patho-aetiological disease processes. This issue might apply in particular to the associations between later puberty timing and asthma and malabsorption/coeliac disease. Similarly, we were unable to distinguish between the potential confounding and mediating effects of body composition and SEP, due to lack of repeated measures of these factors before and after pubertal ages. It is likely that bi-directional causal relationships exist between puberty timing and both body size and SEP. For example, early childhood rapid growth and overweight precede early puberty timing in both sexes^10^,^22^, but in turn early puberty timing leads to subsequent rapid gains in weight and adiposity during adolescence and early adulthood^23^, and with earlier cessation of growth and shorter adult height in women^24^. Similarly, environmental stressors may precede early puberty^25^, but in turn early puberty leads to more risk taking behaviours and poor school performance^8^,^26^,^27^. Hence, we consider that our adjusted estimates are corrected for mediation as well as for potential confounding, and therefore they may underestimate the true nature of the disease relationships with puberty timing. Finally, our consideration of a wide range of potential adverse health outcomes raised the possibility of spurious associations arising out of multiple testing. To minimize this possibility, our approach was to set a highly conservative statistical significance threshold, which was probably over-conservative as many of the tests that we performed are not independent. Future analyses should focus on specific disease categories identified through prospective follow-up, and substantiated by objective external case-verification.

Our study has a number of strengths, notably the very large sample size, which allowed not only more accurate precision of the effect estimates, but also higher resolution on the shape of the observed associations. Our consideration of non-linear associations greatly increased the number of implicated health outcomes, revealing a range of diseases associated with both early and late puberty timing. While linear models appear to be appropriate for some outcomes, such as osteoporosis and breast cancer, both ends of the puberty timing spectrum are likely to have adverse consequences for other later life health outcomes. Our findings are consistent with those of a recent systematic review^7^, which summarised that earlier menarche was associated with higher risk of all-cause mortality, while later menarche was not protective, and some individual reports commented even on the apparent higher mortality in women with late menarche. Notably, our separate consideration of early and late menarche revealed robust associations with higher risks for early natural menopause in women. In contrast, previous studies and reviews focussed on the continuous relationship between these two reproductive variables. In a recent comprehensive review, Forman *et al.* found that of 36 published studies, ten reported a significant direct association, two reported an inverse association, and the remainder reported null findings^8^. Our findings indicate the presence of multiple life-course biological pathways to early menopause.

In summary, this large-scale systematic assessment robustly confirms the associations between early puberty timing and T2D/cardiovascular disease in women and demonstrates concordant associations with early puberty timing in men. Furthermore, the findings implicate links between puberty timing across a broad range of disease associations in men and women, many reported for the first time. Our findings do not indicate that avoidance or treatment of abnormal puberty timing will invariably have widespread beneficial consequences^28^, but rather further work is needed to understand the possible psychosocial, adiposity-related and adiposity-independent mechanisms that link puberty timing to later life health outcomes.

## Additional Information

**How to cite this article**: Day, F. R. *et al.* Puberty timing associated with diabetes, cardiovascular disease and also diverse health outcomes in men and women: the UK Biobank study. *Sci. Rep.* **5**, 11208; doi: 10.1038/srep11208 (2015).

## Supplementary Material

Supplementary Information

## Figures and Tables

**Table 1:** Statistically significant disease/adverse health outcome associations with age at menarche in women.

	Early Menarche (8–11 years)				Late Menarche (15–19 years)					
	Unadjusted		Adjusted		Unadjusted		Adjusted		Linear Trend	
Disease	OR	P	OR	P	OR	P	OR	P	P	P adj.
***Cancer***										
Breast cancer	1.09 (1.03-1.16)	0.002	**1.13 (1.06-1.20)**	**5.6E-05**	0.94 (0.88-1.00)	0.04	0.94 (0.88-1.00)	0.05	**4.3E-08**	**9.2E-11**
Cervical cancer	1.20 (1.05-1.38)	0.008	1.09 (0.95-1.25)	0.22	**1.37 (1.19-1.58)**	**1.2E-05**	1.24 (1.07-1.43)	0.003	0.06	0.07
Uterine/Endometrial cancer	1.39 (1.17-1.64)	1.5E-04	1.23 (1.04-1.46)	0.02	0.99 (0.81-1.20)	0.91	0.99 (0.81-1.20)	0.91	**4.3E-06**	0.002
***Cardiovascular/Metabolic/Endocrine***										
Angina	**1.54 (1.41-1.68)**	**<1E-21**	**1.23 (1.13-1.34)**	**4.6E-06**	**1.39 (1.27-1.52)**	**4.0E-12**	1.20 (1.09-1.32)	1.3E-04	0.02	0.84
Deep venous thrombosis (DVT)	**1.31 (1.21-1.42)**	**1.8E-11**	1.15 (1.06-1.25)	5.4E-04	**1.19 (1.09-1.3)**	**6.1E-05**	1.16 (1.06-1.26)	7.8E-04	0.002	0.71
Heart attack/Myocardial infarction	**1.42 (1.25-1.61)**	**1.0E-07**	1.17 (1.02-1.33)	0.02	1.3 (1.13-1.49)	2.3E-04	1.11 (0.97-1.28)	0.13	0.14	0.50
High cholesterol	**1.23 (1.18-1.28)**	**<1E-21**	1.06 (1.02-1.11)	0.006	**1.09 (1.05-1.14)**	**6.3E-05**	1.02 (0.98-1.07)	0.30	**2.3E-11**	0.009
Hypertension	**1.37 (1.33-1.41)**	**<1E-21**	**1.13 (1.1-1.16)**	**1.6E-15**	0.96 (0.93-0.99)	0.006	0.96 (0.93-0.99)	0.008	**<1E-21**	**<1E-21**
Hypothyroidism	**1.15 (1.10-1.20)**	**4.9E-10**	1.04 (1.00-1.09)	0.06	1.03 (0.98-1.08)	0.20	1.02 (0.97-1.07)	0.49	**3.3E-07**	0.22
Obesity†	**1.91 (1.86-1.96)**	**<1E-21**	**1.82 (1.77-1.87)**	**<1E-21**	**0.85 (0.82-0.88)**	**<1E-21**	**0.76 (0.74-0.79)**	**<1E-21**	**<1E-21**	**<1E-21**
Pulmonary embolism +/- DVT	**1.29 (1.14-1.46)**	**6.9E-05**	1.13 (0.99-1.28)	0.06	1.19 (1.04-1.36)	0.01	1.15 (1-1.31)	0.05	0.09	0.94
Type 2 diabetes	**1.76 (1.62-1.91)**	**<1E-21**	**1.25 (1.15-1.36)**	**5.2E-07**	0.94 (0.85-1.04)	0.22	0.90 (0.81-1.0)	0.05	**<1E-21**	**2.1E-11**
***Gynaecological /Obstetric***										
Early menopause	**1.41 (1.33-1.5)**	**<1E-21**	**1.36 (1.28-1.45)**	**<1E-21**	**1.25 (1.17-1.33)**	**8.6E-12**	**1.16 (1.09-1.24)**	**6.1E-06**	6.0E-03	2.0E-05
Endometriosis	1.19 (1.08-1.31)	2.3E-04	1.16 (1.06-1.28)	0.001	0.89 (0.80-0.99)	0.03	0.90 (0.81-1.00)	0.05	**3.2E-08**	**6.5E-07**
Preeclampsia	1.11 (0.97-1.27)	0.11	1.05 (0.92-1.2)	0.50	0.83 (0.72-0.97)	0.02	0.88 (0.75-1.02)	0.10	**4.0E-05**	0.01
Hysterectomy	**1.33 (1.27-1.39)**	**<1E-21**	**1.25 (1.19-1.31)**	**<1E-21**	1.10 (1.05-1.16)	1.4E-04	1.03 (0.98-1.09)	0.23	**<1E-21**	**<1E-21**
Low birthweight of child	**1.11 (1.06-1.16)**	**2.2E-05**	1.03 (0.99-1.09)	0.17	**1.15 (1.10-1.21)**	**2.0E-08**	1.07 (1.02-1.13)	0.006	0.20	0.27
Oopherectomy	**1.34 (1.28-1.40)**	**<1E-21**	**1.25 (1.19-1.30)**	**<1E-21**	1.10 (1.04-1.15)	1.7E-04	1.05 (1.00-1.10)	0.04	**<1E-21**	**<1E-21**
Still birth	**1.21 (1.12-1.31)**	**2.5E-06**	1.11 (1.03-1.20)	0.01	1.17 (1.08-1.27)	2.3E-04	1.07 (0.98-1.17)	0.12	0.70	0.60
Uterine fibroids	**1.17 (1.10-1.26)**	**4.8E-06**	**1.16 (1.08-1.25)**	**2.3E-05**	**0.85 (0.78-0.92)**	**6.3E-05**	0.85 (0.79-0.92)	9.6E-05	**<1E-21**	**<1E-21**
***Gastrointestinal disorder***										
Allergy to food	**1.41 (1.21-1.65)**	**1.0E-05**	**1.39 (1.19-1.62)**	**3.7E-05**	1.08 (0.91-1.28)	0.40	1.12 (0.95-1.34)	0.18	1.3E-03	0.02
Cholelithiasis/Gall stones	**1.38 (1.28-1.48)**	**<1E-21**	1.16 (1.08-1.25)	1.2E-04	0.91 (0.84-1)	0.04	0.89 (0.82-0.98)	0.01	**<1E-21**	**2.5E-10**
Diverticular disease/Diverticulitis	1.15 (1.04-1.27)	0.005	1.04 (0.94-1.14)	0.48	0.89 (0.80-0.99)	0.04	0.85 (0.76-0.95)	0.004	**6.2E-07**	2.4E-04
Gastric reflux	**1.27 (1.19-1.34)**	**5.8E-15**	1.10 (1.04-1.17)	0.002	**1.15 (1.08-1.22)**	**2.2E-05**	1.08 (1.01-1.15)	0.02	3.0E-04	0.35
Hiatus hernia	**1.41 (1.30-1.51)**	**<1E-21**	**1.19 (1.1-1.28)**	**7.9E-06**	1.16 (1.07-1.26)	3.5E-04	1.09 (1.00-1.18)	0.05	**1.4E-07**	0.03
Irritable bowel syndrome	**1.19 (1.11-1.27)**	**2.4E-07**	1.14 (1.07-1.22)	**7.1E-05**	1.07 (1-1.15)	0.06	1.02 (0.95-1.1)	0.51	1.0E-04	9.7E-05
Malabsorption/Coeliac disease	0.87 (0.73-1.03)	0.11	0.95 (0.8-1.13)	0.55	**1.69 (1.45-1.98)**	**1.9E-11**	**1.62 (1.39-1.89)**	**8.6E-10**	**<1E-21**	**4.3E-12**
***Musculoskeletal disorder***										
Arthritis (nos)	**1.35 (1.19-1.52)**	**2.5E-06**	1.20 (1.06-1.36)	0.005	0.99 (0.86-1.14)	0.87	0.94 (0.81-1.09)	0.41	**2.9E-06**	8.7E-04
Cervical spondylosis	**1.34 (1.18-1.53)**	**8.0E-06**	1.20 (1.05-1.37)	0.007	1.31 (1.14-1.51)	1.3E-04	1.16 (1.01-1.33)	0.04	0.37	0.35
Osteoporosis	**0.84 (0.78-0.91)**	**7.1E-06**	0.91 (0.84-0.98)	0.02	**1.17 (1.09-1.26)**	**2.9E-05**	1.09 (1.01-1.18)	0.03	**<1E-21**	**2.5E-06**
Osteoarthritis	**1.31 (1.26-1.37)**	**<1E-21**	**1.14 (1.09-1.18)**	**2.7E-10**	1.06 (1.02-1.11)	0.006	1.03 (0.98-1.08)	0.20	**<1E-21**	**2.8E-08**
Rheumatoid arthritis	**1.24 (1.12-1.37)**	**1.9E-05**	1.14 (1.03-1.26)	0.01	**1.27 (1.15-1.41)**	**6.2E-06**	1.17 (1.05-1.3)	0.004	0.43	0.40
Short stature	**1.58 (1.50-1.67)**	**<1E-21**	**1.19 (1.09-1.30)**	**1.2E-05**	0.96 (0.91-1.02)	0.24	**0.80 (0.72-0.89)**	**2.4E-05**	**<1E-21**	**<1E-21**
Spine arthritis/Spondylitis	**1.50 (1.33-1.68)**	**1.0E-11**	**1.32 (1.17-1.48)**	**4.6E-06**	1.16 (1.02-1.32)	0.03	1.04 (0.91-1.19)	0.56	**9.9E-06**	1.7E-04
***Neuro/Cognition/Psychiatry***										
Depression	**1.19 (1.14-1.25)**	**1.1E-13**	1.05 (1.01-1.10)	0.03	**1.12 (1.07-1.18)**	**6.3E-06**	1.07 (1.02-1.13)	0.008	0.007	0.36
Low intelligence (Fluid)	1.14 (1.01-1.28)	0.03	0.94 (0.83-1.06)	0.34	**1.76 (1.57-1.97)**	**<1E-21**	**1.32 (1.18-1.49)**	**2.4E-06**	**<1E-21**	**2.0E-9**
***Respiratory/ENT disorder***										
Asthma	**1.17 (1.13-1.21)**	**<1E-21**	1.06 (1.03-1.1)	8.3E-04	**1.10 (1.06-1.14)**	**9.4E-07**	**1.11 (1.07-1.15)**	**7.9E-08**	1.6E-04	0.02
Emphysema/Chronic bronchitis	**1.35 (1.21-1.5)**	**5.3E-08**	1.18 (1.05-1.31)	0.004	**1.50 (1.35-1.68)**	**5.7E-13**	1.23 (1.1-1.38)	2.7E-04	0.004	0.11
Low FEV1	1.07 (1.01-1.13)	0.02	1 (0.95-1.06)	0.90	**1.2 (1.14-1.28)**	**4.81E-10**	1.1 (1.04-1.17)	0.001	**4.4E-13**	0.02
***Other***										
Glaucoma	1.24 (1.1-1.4)	4.9E-04	1.23 (1.09-1.39)	0.001	0.96 (0.83-1.1)	0.52	0.98 (0.85-1.12)	0.72	**3.1E-05**	2.9E-04
Poor sleep	**1.59 (1.45-1.75)**	**<1E-21**	**1.30 (1.18-1.43)**	**1.3E-07**	**1.48 (1.35-1.64)**	**3.3E-15**	**1.25 (1.13-1.38)**	**2.0E-05**	0.03	0.16
Poor overall health	**1.51 (1.42-1.60)**	**<1E-21**	1.10 (1.03-1.18)	0.003	**1.33 (1.25-1.43)**	**<1E-21**	**1.19 (1.11-1.27)**	**1.3E-06**	**8.2E-07**	0.08
Psoriasis	**1.29 (1.15-1.45)**	**2.1E-05**	1.20 (1.07-1.35)	0.002	1.02 (0.89-1.16)	0.82	0.99 (0.86-1.12)	0.82	4.9E-04	0.007

Multiple test-corrected statistically significant (P-value < 7.48×10^−5^) associations are highlighted in bold. Early/Late menarche groups are compared to the median group (menarche at age 13 years). Baseline models included birth year, age and age^2^; Adjusted models also included principle components for socioeconomic position and adiposity/body composition, †except where ‘Obesity’ was the outcome, in which case the adjusted models included only principle components for socioeconomic position.

**Table 2:** Statistically significant disease/adverse health outcome associations with relative age at voice breaking in men.

	Early voice breaking				Late voice breaking			
	Unadjusted		Adjusted		Unadjusted		Adjusted	
Disease	OR	P	OR	P	OR	P	OR	P
***Cardiovascular/Metabolic/Endocrine***								
Angina	**1.44 (1.31-1.6)**	**5.2E-13**	**1.39 (1.25-1.54)**	**2.7E-10**	0.8 (0.72-0.9)	9.0E-05	0.94 (0.84-1.05)	0.29
Gout	**1.29 (1.14-1.46)**	**4.8E-05**	1.16 (1.03-1.32)	0.02	0.85 (0.75-0.97)	0.01	1.01 (0.89-1.15)	0.89
Heart attack	**1.32 (1.18-1.47)**	**7.9E-07**	**1.26 (1.13-1.41)**	**3.5E-05**	**0.75 (0.67-0.85)**	**2.9E-06**	0.88 (0.78-0.99)	0.04
High cholesterol	**1.2 (1.12-1.27)**	**1.6E-08**	1.13 (1.06-1.21)	1.3E-04	**0.84 (0.79-0.89)**	**5.8E-09**	0.93 (0.87-0.99)	0.02
Hypertension	**1.25 (1.19-1.31)**	**<1E-21**	**1.11 (1.06-1.17)**	**4.4E-05**	**0.77 (0.74-0.81)**	**<1E-21**	0.91 (0.87-0.96)	1.7E-04
Hypothyroidism/Myxoedema	**1.38 (1.18-1.62)**	**6.7E-05**	1.32 (1.12-1.55)	7.2E-04	1.07 (0.92-1.24)	0.41	1.17 (1-1.36)	0.05
Obesity†	**1.52 (1.45-1.6)**	**<1E-21**	**1.58 (1.50-1.66)**	**<1E-21**	**0.58 (0.55-0.61)**	**<1E-21**	**0.62 (0.59-0.65)**	**<1E-21**
Type 2 diabetes	**1.44 (1.3-1.59)**	**6.8E-13**	**1.24 (1.11-1.37)**	**6.7E-05**	**0.69 (0.61-0.77)**	**2.2E-10**	0.87 (0.77-0.98)	0.03
***Neuro/Psychiatry***								
Anxiety/Panic attacks	1.24 (1.02-1.5)	0.03	1.22 (1.01-1.48)	0.04	**1.42 (1.21-1.66)**	**1.3E-05**	**1.43 (1.22-1.67)**	**9.3E-06**
Bipolar disorder	**2.05 (1.5-2.8)**	**6.2E-06**	1.75 (1.28-2.4)	5.2E-04	1.63 (1.21-2.2)	0.001	1.62 (1.2-2.19)	0.002
Chronic fatigue syndrome	1.54 (1.08-2.2)	0.02	1.50 (1.05-2.15)	0.03	**1.92 (1.45-2.54)**	**5.1E-06**	1.77 (1.33-2.35)	7.8E-05
Depression	**1.37 (1.24-1.5)**	**1.1E-10**	**1.28 (1.16-1.41)**	**6.2E-07**	**1.29 (1.19-1.4)**	**2.7E-09**	**1.36 (1.25-1.49)**	**1.1E-12**
***Other***								
Asthma	1.14 (1.07-1.22)	8.3E-05	1.11 (1.04-1.19)	0.002	**1.22 (1.16-1.29)**	**2.2E-12**	**1.22 (1.16-1.29)**	**3.6E-12**
Eczema/Dermatitis	1.06 (0.93-1.22)	0.36	1.06 (0.93-1.21)	0.41	**1.38 (1.25-1.53)**	**8.8E-10**	**1.30 (1.18-1.45)**	**5.4E-07**
Irritable bowel syndrome	**1.47 (1.25-1.73)**	**3.3E-06**	**1.49 (1.26-1.75)**	**2.2E-06**	**1.37 (1.19-1.59)**	**1.8E-05**	1.34 (1.15-1.55)	1.0E-04
Poor sleep	**1.99 (1.69-2.33)**	**<1E-21**	**1.94 (1.63-2.30)**	**2.1E-14**	0.89 (0.74-1.07)	0.23	1.08 (0.89-1.30)	0.45
Poor overall health	**1.58 (1.45-1.72)**	**<1E-21**	**1.44 (1.31-1.58)**	**7.6E-15**	1.00 (0.91-1.09)	0.99	**1.25 (1.13-1.37)**	**4.1E-06**
Short stature	**1.45 (1.33-1.58)**	**<1E-21**	**1.39 (1.24-1.56)**	**1.6E-08**	1.03 (0.95-1.12)	0.45	1.00 (0.89-1.11)	0.95

Multiple test-corrected statistically significant (P-value < 7.48 × 10^−5^) associations are highlighted in bold. Early/Late voice breaking groups are compared to the group who reported voice breaking at “about normal age”. Baseline models included birth year, age and age^2; Adjusted models also included principle components for socioeconomic position and adiposity/body composition, †except where ‘Obesity’ was the outcome, in which case the adjusted models included only principle components for socioeconomic position.
